# Modeling radio-frequency energy-induced heating due to the presence of transcranial electric stimulation setup at 3T

**DOI:** 10.1007/s10334-020-00853-5

**Published:** 2020-05-27

**Authors:** Mikhail Kozlov, Marc Horner, Wolfgang Kainz, Nikolaus Weiskopf, Harald E. Möller

**Affiliations:** 1 Max Planck Institute for Human Cognitive and Brain Sciences, Stephanstrasse 1a, 04103 Leipzig, Germany; 2 ANSYS, Inc., 1007 Church Street, Suite 250, Evanston, IL 60201 USA; 3 Office of Science and Engineering Laboratories, Division of Biomedical Physics, U.S. FDA, CDRH, Silver Spring, MD 20993 USA; 4 Felix Bloch Institute for Solid State Physics, Faculty of Physics and Earth Sciences, Leipzig University, Linnéstrasse 5, 04103 Leipzig, Germany

**Keywords:** Computational modeling, RF simulations, Tissue heating, Transcranial direct current stimulation, Finite element method (FEM)

## Abstract

**Purpose:**

The purpose of the present study was to develop a numerical workflow for simulating temperature increase in a high-resolution human head and torso model positioned in a whole-body magnetic resonance imaging (MRI) radio-frequency (RF) coil in the presence of a transcranial electric stimulation (tES) setup.

**Methods:**

A customized human head and torso model was developed from medical image data. Power deposition and temperature rise (Δ*T*) were evaluated with the model positioned in a whole-body birdcage RF coil in the presence of a tES setup. Multiphysics modeling at 3T (123.2 MHz) on unstructured meshes was based on RF circuit, 3D electromagnetic, and thermal co-simulations. Δ*T* was obtained for (1) a set of electrical and thermal properties assigned to the scalp region, (2) a set of electrical properties of the gel used to ensure proper electrical contact between the tES electrodes and the scalp, (3) a set of electrical conductivity values of skin tissue, (4) four gel patch shapes, and (5) three electrode shapes.

**Results:**

Significant dependence of power deposition and Δ*T* on the skin’s electrical properties and electrode and gel patch geometries was observed. Differences in maximum Δ*T* (> 100%) and its location were observed when comparing the results from a model using realistic human tissue properties and one with an external container made of acrylic material. The electrical and thermal properties of the phantom container material also significantly (> 250%) impacted the Δ*T* results.

**Conclusion:**

Simulation results predicted that the electrode and gel geometries, skin electrical conductivity, and position of the temperature sensors have a significant impact on the estimated temperature rise. Therefore, these factors must be considered for reliable assessment of Δ*T* in subjects undergoing an MRI examination in the presence of a tES setup.

## Introduction

Simultaneous magnetic resonance imaging (MRI) with transcranial electric stimulation (tES) or electroencephalography (EEG) are promising non-invasive techniques for the study of human brain function [1–5]. Usually, tES is applied in the form of either transcranial direct current stimulation (tDCS) or transcranial alternating current stimulation (tACS). Simultaneous tDCS and magnetic resonance (MR) experiments have been used, for example, to: (1) evaluate tissue metabolite changes in the human motor cortex immediately following tDCS [6], (2) validate modulation of ventro-medial prefrontal cortex activity [7], (3) investigate the modulatory stimulation effects underlying behavioral improvements on resting-state activity and connectivity [8] or task-related activity and effective connectivity [9], and (4) analyze the neural mechanisms underlying behavioral tDCS effects with high spatial resolution across the entire brain [10]. Combining tACS with simultaneous fMRI has shown that the stimulation effects are state-, current-, and frequency-dependent, and that modulation of brain activity is not limited to the area directly below the electrodes [11].

Combined EEG and tES setups consist of electrodes located in close proximity to the human skin, electrical wires that connect the electrodes to a control unit, and a high-conductivity gel that ensures good electrical contact between the electrodes and the skin. The wires enter the effective exposure volume of the radio-frequency (RF) coil, operating as an antenna. An electric current produced at an  EEG or tES electrode depends on the relative positioning of the wires and the human body, the electrical contact of the electrode with the skin, and the tissue structure(s). A local temperature increase (Δ*T*) in the tissue may result either directly from RF energy deposition, or indirectly from contact with the electrodes and gel, that are themselves heated due to RF energy deposition. Δ*T* of human tissue was identified as a safety concern for subjects undergoing multi-modal MR examinations in the International Electrotechnical Commission Standard (IEC) 60601-2-33 [12].

Measurements of Δ*T* during MR experiments in the presence of EEG or tES setups have been obtained experimentally [13–23]. In the vast majority of these investigations different types of fiber-optic temperature (FOT) probes were used [13–21]. The absence of conductive materials in the MR environment results in negligible interaction between the electromagnetic (EM) field of the MR scanner and FOT probes. Thermocouples were used in some studies [20, 23] despite reported measurement errors of hard-wire thermistor or thermocouple-based sensors, due to interference from the MR scanner’s EM emissions [24]. Several electrode arrangements have been investigated using these approaches, including placement: (1) on a container made of dielectric material and filled with a tissue-simulating medium [16, 18]; (2) on human skin [14–18, 23]; (3) on a conductive gel layer covering a dielectric mold with a realistic head shape and filled with agar gel [19, 20]; (4) on a solid gel phantom with a realistic head shape and an electrical conductivity typical of human head tissue (0.6–1 S/m) [20, 21]; (5) on watermelons of similar size to the human head [15]; and (6) inside a gel [13]. Conductive gel was sometimes used to ensure a similar setup as in human studies. Temperature sensors were positioned primarily underneath the electrode, or as close as possible, to evaluate Δ*T* for a given RF exposure condition.

Δ*T* depends directly on the RF exposure conditions, i.e., the employed MR examination protocol, and is reported to vary with the number of electrodes, the root mean square of the generated RF field, RF coil geometry, size of the human subject, and the electrical properties of the tissues [18, 20, 21]. In addition, variation in the locations of the temperature probes, the probes’ sensitive element dimensions, and the protective coating of the FOT sensors, limit straightforward comparisons of Δ*T* values reported in different studies. As an example, despite having similar setups, relatively small Δ*T* values (< 3 °C) were observed in some experiments (e.g., [17, 20]), whereas others reported relatively high Δ*T* values (> 8 °C) [18]. Moreover, the measured skin temperatures before RF exposure varied between 18 °C [16] and 36 °C [14].

For EEG electrodes in contact with the human skin, Δ*T* has been investigated numerically [19–21]. However, substantial simplifications were made in the modeling of the MRI coil, the device, and the human subject. For example, Jorge et al. [19] did not model the temperature rise. Angelone et al. [21] reported temperature results on a 3 mm × 3 mm × 3 mm spatial grid, which is substantially larger than the thickness of some human head tissues. Atefi et al. [20] used a two-step approach consisting of modeling the EM field generated by an RF coil on the surface of a cylinder, which was used as a radiating boundary condition on the surface of a container that included a human model and the device of interest. Numerical uncertainty is expected to increase using this approach, depending on the magnitude of the two-way interaction between the coil and the human subject with a device. Atefi et al. [20] also stated that their results were “limited by the absence of a torso in the human body model, which precluded modeling a body coil and the full lead length, possibly also affecting RF-induced currents in the lead”.

In all combined MRI and EEG or tES studies the Δ*T* induced by the RF coil of a given scanner should be evaluated to ensure participant safety. A reliable numerical Δ*T* assessment requires correct modeling of: (1) the scanner’s RF coil with a set of human models at the MR resonance frequency, (2) electrical and thermal contacts between the electrodes and the skin, and (3) the insulated tES wires, which may have helical or twisted geometries. Further, for tES setups, a wire with a length on the order of 1 m runs to the connecting or filter box, which can be located outside the scanner’s magnet bore.

Voxel-based human models with or without electrodes are commonly simulated using time-domain solvers and hexahedral meshes. The hexahedral mesh results in a staircase discretization of the curved surfaces of the RF coil structures, electrodes, human tissues, and helical wires. The mesh resolution should be substantially smaller than the thinnest tissue, the electrode thickness, and the wire diameter to achieve realistic contact properties and wire impedance. These issues, as well as a high quality factor of typical RF coils for MRI, tend to result in long simulation times for this class of solvers. The Δ*T* assessment is also altered when individual mesh elements span multiple tissues. This can occur when a hexahedral mesh is not aligned with the voxels of the human model (see, e.g., the material property maps reported in [25]). Such modification of the material properties increases the uncertainty in the model output for voxel-based human body models.

Modern three-dimensional (3D) solvers based on the finite-element method (FEM) and unstructured meshes are good candidates for multi-physics modeling of multi-modality MR setups for several reasons. First, each tissue object is meshed based on its boundaries. Second, the mesh size can be adjusted individually for each model object, and third, the simulation time depends only slightly on the size of the smallest mesh elements and quality factor of the scanner RF coil. However, it is important that the geometric models of all components are error-free (i.e., no self-intersections, no over-connections, etc.) to generate a successful mesh. The technical challenges associated with creating geometrically consistent models are most likely the primary reason why commercial 3D EM and thermal solvers are less commonly used to study inter-subject Δ*T* variability in subjects during tES setups.

To address this requirement, we have recently developed a semi-automatic processing pipeline to generate individualized surface-based models of the human head and upper torso from MR images of individual subjects [26]. The main goal of the current study was to develop a numerical workflow for modeling the temperature rise in a high-resolution human head and torso model, positioned in a whole-body birdcage RF coil at 3T (123.2 MHz) and in the presence of a tES setup. The developed workflow was used to estimate dependencies of Δ*T* on the shape of the electrodes and gel patches, electrical conductivities of the skin and gel, and skin thickness. We further quantify the variation of Δ*T* for a set of phantoms that could be constructed from synthetic (tissue-mimicking) materials.

## Methods

### Anatomical models in the presence of a tES setup and an MRI RF coil

**Fig. 1:**
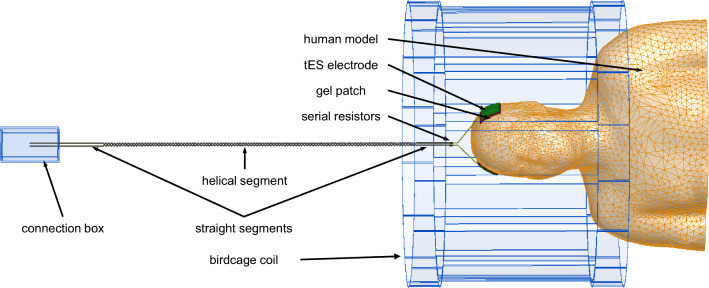
The tES setup attached to the human model located at the head landmark position in a whole-body 3T RF coil

A customized human head and torso model was developed from medical image data obtained in a previous study [26]. A high-resolution human model was positioned at the head landmark position in a whole-body 3T RF coil operating at 123.2 MHz. As in our previous study [27], the RF coil was a 16-rung, high-pass birdcage with an inner diameter of 615 mm and total length of 480 mm, which are common construction parameters for clinical, standard-bore, 3T scanners of a major vendor. The coil was shielded by a metal enclosure that mimicked a 1220 mm-long scanner bore. The tES setup consisted of two electrodes, two gel patches, two leads, and a metal connection box (70 mm × 45 mm × 130 mm) located 410 mm away from the coil enclosure (see Fig. 1). The numerical domain size was defined by an 820 mm × 820 mm × 1920 mm air box with radiation boundaries on all outer faces.

**Fig. 2:**
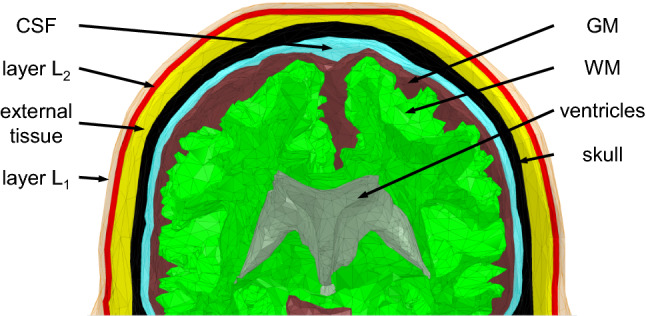
Close-up cross-sectional view of the human head model

**Fig. 3:**
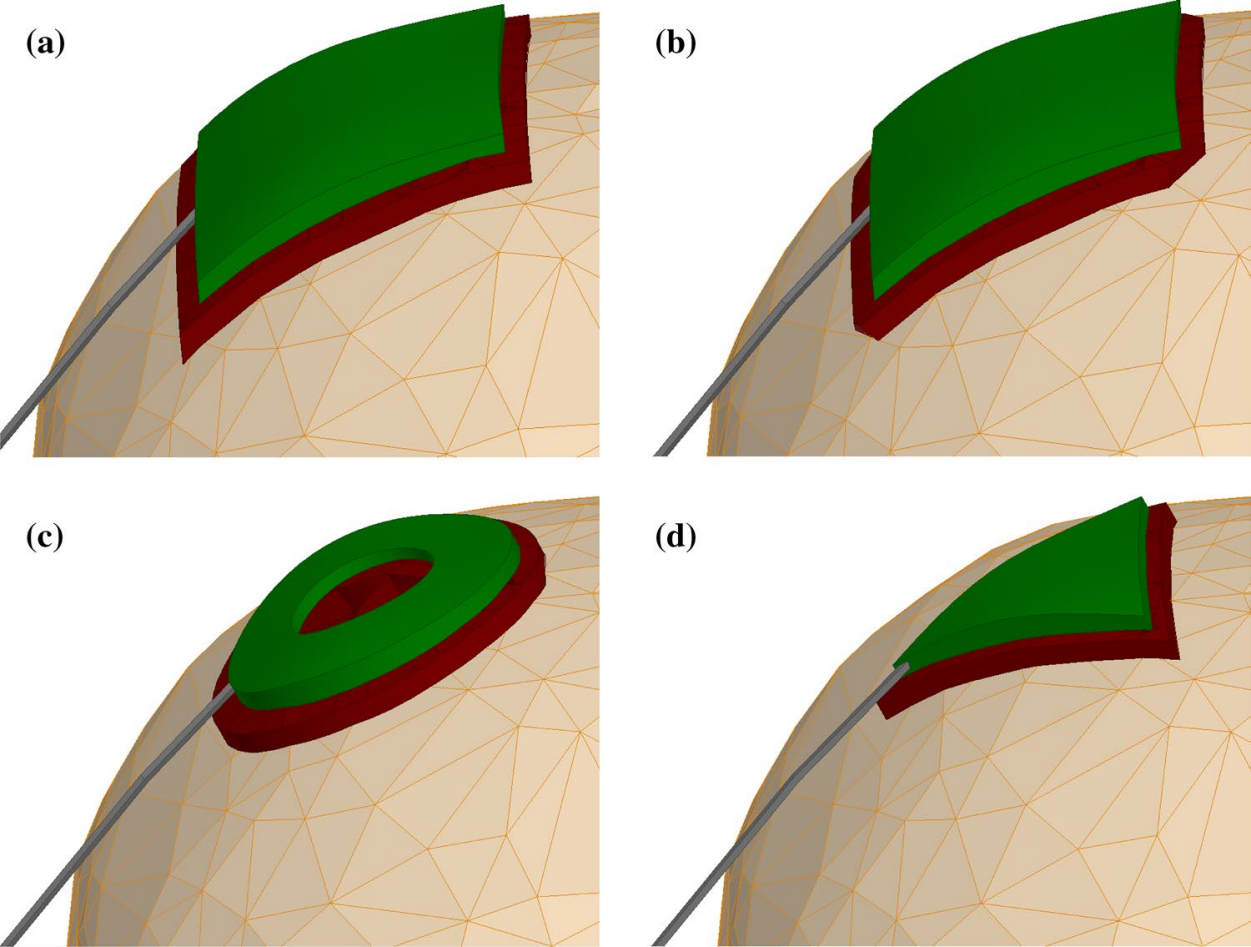
Close-up view of the tES electrode, gel patch, and wire. **a** Square electrode and rectangular gel patch. **b** Square electrode and rectangular gel patch with chamfered corners. **c** Hollow cylinder electrode and cylindrical gel patch. **d** Triangular electrode and triangular gel patch

Our semi-automatic processing pipeline for the generation of subject-specific human head and torso models [26] identified the following structures: torso bones, lungs, skull, air, spinal cord, cerebro-spinal fluid (CSF), ventricles, cerebral gray matter (GM), and white matter (WM) as being relevant for MRI RF safety assessment. An external tissue object was also created as a combination of non-segmented tissue structures, including skin. All objects were defined by external triangulated surfaces. The coarse meshes presented in Figs. 1, 2, and 3 show the geometrical meshes of the human model objects, gel, and electrodes, respectively. A single boundary between adjacent objects eliminated intersections or intermediate gaps. The scalp tissue structure was not segmented in the pipeline due to the limited resolution of the underlying MRI data (which had 1 mm isotropic pixel size).

The scalp is usually described as having five layers: skin, connective tissue, epicranial aponeurosis, loose areolar tissue, and pericranium. Connective tissue consists of a dense subcutaneous layer of fat and fibrous tissue containing the nerves and vessels of the scalp. The skin thickness is correlated with several parameters including race, age, gender, skin type, and skin location [28–32]. A variation of skin thickness at different locations has also been reported [29]. With a total thickness of less than 1 cm, the epicranial, areolar, and pericranial scalp layers are significantly smaller than the electrical wavelengths in human tissues at 123.2 MHz (~ 17 cm). Under these conditions, different tissues with similar electrical properties can be modeled as a single layer with average tissue properties.

In the customized human model, the scalp was modeled using three layers. Two layers, L_1_ and L_2_, with thicknesses *δ*_1_ and *δ*_2_, respectively, were separated from the external tissue object while maintaining the model’s external dimensions (see Fig. 2) using ANSYS SpaceClaim (ANSYS, Inc., Canonsburg, PA, USA). Three scalp model configurations denoted Sc_1_, Sc_2_, and Sc_3,_ were simulated to evaluate the dependence of Δ*T* on scalp modeling. Both *δ*_1_ and *δ*_2_ were 2 mm. This value was within a range reported in [31] and [28]. Material property assignments for each configuration were as follows: (1) Sc_1_: L_1_ as skin and L_2_ as fat [28, 33], (2) Sc_2_: L_1_ and L_2_ as skin to investigate a thick-skin scenario that was observed in [32], and (3) Sc_3_: L_1_ as skin and L_2_ as muscle [31]. Since the properties of muscle are similar to those of the combined tissues, the electrical and thermal properties of the external tissue object were assigned the properties of muscle. The electrical and thermal properties of the human tissues in this study were adopted from the IT’IS database [34].

Gabriel et al. [35] reported that the electrical conductivity (*σ*) of the skin can be as small as 0.5 S/m and as high as 1.5 S/m at 100 MHz. The high conductivity electrode gel typical for tES applications can increase the skin conductivity locally. Therefore, the dependence of the Δ*T* results on skin conductivity was investigated for *σ* = {0.5, 0.75, 1.0, 1.25, and 1.5} S/m, while maintaining the thermal properties and relative permittivity (*ε*_r_).

The tES setup was similar to the setup reported in [9, 11]. Each tES electrical wire consisted of one helical and several straight segments. Diameters of the inner copper wire and wire insulator were 1.2 mm and 2.2 mm, respectively. The helical wire pitch was 12.5 mm. A serial resistor of 10 kΩ integrated in each wire was located 100 mm away from the electrodes. The wires were aligned with the axis of the scanner bore as this configuration resulted in the smallest interference of the tES setup and MRI scanning during past measurements [9].

Most simulations were performed for flexible square tES electrodes with edge lengths of 50 mm, which corresponds to dimensions used in previous experimental studies [9–11]. The modeled thickness of the tES electrodes was 3 mm larger than the thickness of the realistic tES electrodes. The thickness was increased to ensure proper electrical connection of the tES wire with the electrode in the numerical model. The electrode material properties (see Table 1) were adopted from properties of a carbon-containing material. A gel patch of approximately 3 mm thickness positioned between the tES electrode and the skin was used to mimic experimental conditions. Two gel patch geometries for square electrodes were investigated: rectangular and rectangular with chamfered corners (Fig. 3a, b). Three types of commercial gels were modeled: ECI electro-gel for electro-caps (Electro-Cap International, Inc., Eaton, OH, USA), Abralyt HiCL (Brainbox Ltd, Cardiff, United Kingdom), and Abralyt2000 (EASYCAP GmbH, Herrsching, Germany). They are labeled as “gel I”, “gel II”, and “gel III”, respectively.

**Table 1:** Material properties used in the simulations

Component	Density kg/m^3^	Electrical conductivity S/m	Relative permittivity	Thermal conductivity W/m/K	Specific heat capacity J/kg/K
Acrylic material	1180	0.55 × 10^–6^	3.14	0.2	1780
c_s_ material	2250	0.518	4	0.37	3391
c_t_ material	2250	0.518	4	24	709
tDCS Electrode	2250	7.0 × 10^4^	1	24	709
Gel I	1001	9.6	45	0.6	4181
Gel II	1001	7.5	50	0.6	4181
Gel III	1001	2.2	73	0.6	4181
Copper wire	8300	5.8 × 10^7^	1	401	385
tDCS wire insulator	1350	0.22 × 10^–8^	2.1	0.2	1000
Skin tissue	1109	0.518	66.5	0.37	3391
Fat tissue	911	0.0695	12.4	0.21	2348
Muscle tissue	1090	0.717	63.8	0.49	3421

Hollow cylinder and equilateral triangle electrode shapes have been utilized in some previous tES studies (without MRI) and were therefore modeled here for completeness (Fig. 3c, d). The outer and inner diameters of the hollow cylinder electrodes were 48 mm and 24 mm, respectively. The side length of the equilateral triangle was 45 mm. Gel patches for the hollow cylinder electrodes were cylindrical in shape with a diameter of 53 mm. Gel patches for the triangle electrodes were equilateral triangles with side lengths of 51 mm. Similar to the square electrodes, the thickness of the gel patches was 3 mm. Hollow cylinder and equilateral triangle electrode shapes were applied to only one anatomical model with L_1_ as skin with 0.5 S/m, L_2_ as fat, and “gel I”.

The following convention (i.e., $\text A \times \text M_{{\text L_{1} }} \times \text M_{{\text L_{2} }} \times \text P_{{{\text{shape}}}} \times \text G$) was used to denote the various modeling configurations for the anatomical model in the presence of the tES setup. “A” was a label for the anatomical models. “$\text M_{{\text L_{1} }}$” and “$\text M_{{\text L_{2} }}$” denoted the materials assigned to layers L_1_ and L_2_, respectively. “P_shape_” was a code for the patch shape, (“r” = rectangular, “c” = rectangular with chamfered corners, “i” = circular, and “t” = triangular). “G” was a code for the gel, where 1 referred to “gel I”, 2 to “gel II”, and 3 to “gel III”. Tissue labels were as follows: “f”, “m”, “s” were used to represent fat, muscle, and skin, respectively. The electrical conductivities of skin (*s*_0.5_, *s*_0.75_, *s*_1_, *s*_1.25_, *s*_1.5_) were equal to 0.5, 0.75, 1.0, 1.25, 1.5 S/m, respectively. For example, A × s_1.5_ × f × r × 1 denoted an anatomical model with L_1_ as skin with 1.5 S/m, L_2_ as fat, rectangular gel patch, and “gel I”. The simulation without the tES setup, i.e. A × s × f, was used as a reference. To see the effect of the cabling on the results, the human model with square electrodes and rectangular gel patches, but without tES wires and the box (see below), was modeled. This model was labeled as E × s_0.5_ × f × r × 1.

### Experimental phantoms in the presence of a tES setup and an MRI RF coil

An experimental phantom typically includes an enclosure made of a rigid material for mechanical stability that is used as a container for the internal tissue simulating media. Until recently, solid gel phantoms without an enclosure were reported only with homogeneous electrical properties [20, 21]. However, progress in 3D printing technology is expected to enable the printing of anatomically accurate phantoms, i.e., experimental phantoms consisting of materials with properties corresponding to those of human tissues. We investigated this scenario as a potential means to validate numerical predictions under conditions that are close to those encountered during the scanning of human subjects. However, blood perfusion and metabolic heat were not taken into consideration.

The accurate experimental phantoms were modeled using the geometrical objects of our customized human head and torso model. The human model simulation results were also applicable to the anatomically accurate phantom because our simulations of human models did not include blood perfusion and metabolic heat. Variations of accurate experimental phantom were defined by changing the electrical and thermal properties of *L*_1_ or *L*_2_.

The following convention, i.e., “$\text P \times \text M_{{\text L_{1} }} \times \text M_{{\text L_{2} }} \times \text P_{{{\text{shape}}}} \times \text G$”, denoted various modeling configurations of experimental phantom in the presence of a tES setup. “*P*” was a label for the experimental phantoms. “$\text M_{{\text L_{1} }}$” and “$\text M_{{\text L_{2} }}$” denoted materials assigned to layer L_1_ and L_2,_ respectively, where “s” was skin, “f” was fat, and “m” was muscle tissue, “a” was acrylic material, “c_t_” was a carbon material with high thermal conductivity, and “c_s_” was a carbon material with thermal properties identical to skin tissue. “P_shape_” and “G” were the same codes as used for the anatomical models.

Four configurations of a phantom enclosure built from an acrylic material and a carbon containing material were investigated: “P × a × f × r × 1”, “P × c_s_ × f × r × 1”, “P × c_t_ × f × r × 1”, and “P × s_0.5_ × a × r × 1”. The material c_s_ was a carbon-containing material with *σ* = 0.5 S/m (similar to that of skin), *ε*_r_ = 4 (significantly smaller than that of skin, *ε*_r_ of 66.5), and thermal properties corresponding to those of skin tissue. The material c_t_ was a carbon-containing material with *σ* = 0.5 S/m, *ε*_r_ = 4, and a high thermal-conductivity material usually observed in carbon-containing materials (see Table 1).

Single-tissue solid gel phantoms without a phantom enclosure were investigated with the rectangular patch shape and “gel I”. The homogeneous gel thermal properties and *ε*_r_ were identical to the properties of skin. Three electrical conductivities of solid gel were studied for this class of phantoms: 0.47 S/m, 0.52 S/m, and 0.75 S/m. The convention “P × SG × r × 1” denotes the examined modeling configurations, where the solid gel label “SG” is denoted as *s*_0.47_, *s*_0.52_, *s*_0.75_ for *σ* = {0.47, 0.52, 0.75} S/m, respectively.

### Model analysis and convergence

The multi-physics evaluation was based on RF-circuit, 3D EM, and thermal co-simulation. We used Keysight ADS (Keysight, Santa Clara, CA, USA) as the circuit simulator, ANSYS HFSS (ANSYS, Canonsburg, PA, USA) as the 3D EM solver, and ANSYS Non-Linear Thermal (NLT) in ANSYS Mechanical (ANSYS, Canonsburg, PA, USA) as the thermal solver. Note that the ANSYS HFSS and ANSYS NLT solvers are FEM-based analysis tools.

The computational modeling workflow consisted of six major steps. First, the whole-body 3T RF coil loaded with the human model, but without the tES setup, was tuned, matched, decoupled, and excited, as previously described in [27, 36]. Next, a head-averaged specific absorption rate (headSAR) was calculated using the built-in functionality of ANSYS HFSS. The transmit power of the RF coil was then adjusted to achieve a headSAR of 3.2 W/kg. The fourth step involved running RF-circuit and 3D EM co-simulations for the human model in the presence of the tES setup while maintaining the tuning, matching, and decoupling conditions obtained in the first step and the transmit power obtained in the third step. The spatially distributed volume and surface losses from ANSYS HFSS were transferred into ANSYS Mechanical NLT with simultaneous mapping of the losses to the ANSYS NLT mesh grid using ANSYS Workbench (ANSYS, Canonsburg, PA, USA). Finally, these losses were assigned as thermal sources and the transient thermal simulation was performed in ANSYS NLT with continuous input power for a total transient time of 540 s.

The target value for headSAR was 3.2 W/kg, corresponding to the maximum exposure level defined by IEC 60601-2-33 Ed. 3 for a patient at the head landmark position [14]. The whole-body 3T RF coil, tES connection box, and wires between the connection box and serial resistors were not included in the thermal simulations. A thermal radiation boundary condition was defined on all external surfaces of the human model, gel, and electrodes. To mimic the worst-case conditions, the ambient temperature was set to 37 °C and air convection was not taken into account. Since the initial steady-state temperature distributions in the human model and the tES setup were not calculated, the initial temperatures of the gel patch, electrodes, and human tissues were assumed to be 37 °C in all simulations. The direct thermal solver of ANSYS NLT was used. The initial time step of the transient thermal simulation was 1 ms, which is more than ten times shorter than the shortest thermal time constant in the simulation. The flux convergence of the thermal solver was set to 0.0001. Blood perfusion and metabolic heat generation were not taken into account because the built-in user interface of ANSYS NLT does not support the inclusion of temperature-dependent metabolic heat generation and tissue-dependent perfusion.

The primary output of the modeling workflow was Δ*T* at different locations, i.e., not an absolute temperature distribution. The decision to focus on Δ*T* assessment was based on the following considerations. The initial temperatures of skin, gel, and electrodes depend on a number of external factors whose implementation is not trivial in a thermal steady-state model. Specifically, published implementations of a steady-state model for skin temperature were based on: (1) integrating the Pennes’ bioheat equation (that included metabolic heat generation and perfusion), boundary conditions, and initial conditions for a duration until equilibrium was reached [37, 38]; or (2) solving the steady-state bioheat equation [39]. The modeled initial skin temperature reported was 35 °C [37, 39], which is closer to the upper level of the initial skin temperature reported in measurements. Additionally, the RF energy-induced heating due to the presence of a tES setup is localized to a small tissue volume. Since the initial spatial temperature gradient is small in this volume and adjacent regions, the uncertainty in Δ*T,* due to the assumption of a uniform initial temperature throughout the head, is expected to be relatively small.

A noticeable temperature rise due to the presence of a tES setup appeared only in the scalp and skull. Blood perfusion and metabolic heat generation in scalp tissues (not considered in our simulations) are significantly smaller than in transcranial tissues [39]. Thermoregulatory processes show typical response times on the order of 10 min [40]. Murbach et al. [40] modeled thermal hotspots in a human model positioned in a 1.5 T MRI coil with blood perfusion. Reported observations showed that the perfusion thermal regulation significantly influenced temperature rise at the shoulder if the temperature increase was larger than 4 °C and the RF exposure time longer than 8 min. After 10 min of high RF exposure, the observed temperature increase was negligible [40]. These observations, as well as the omission of blood perfusion and metabolic heating, served as the rational for setting the RF exposure time to 540 s to prevent substantial Δ*T* overestimation.

**Fig. 4:**
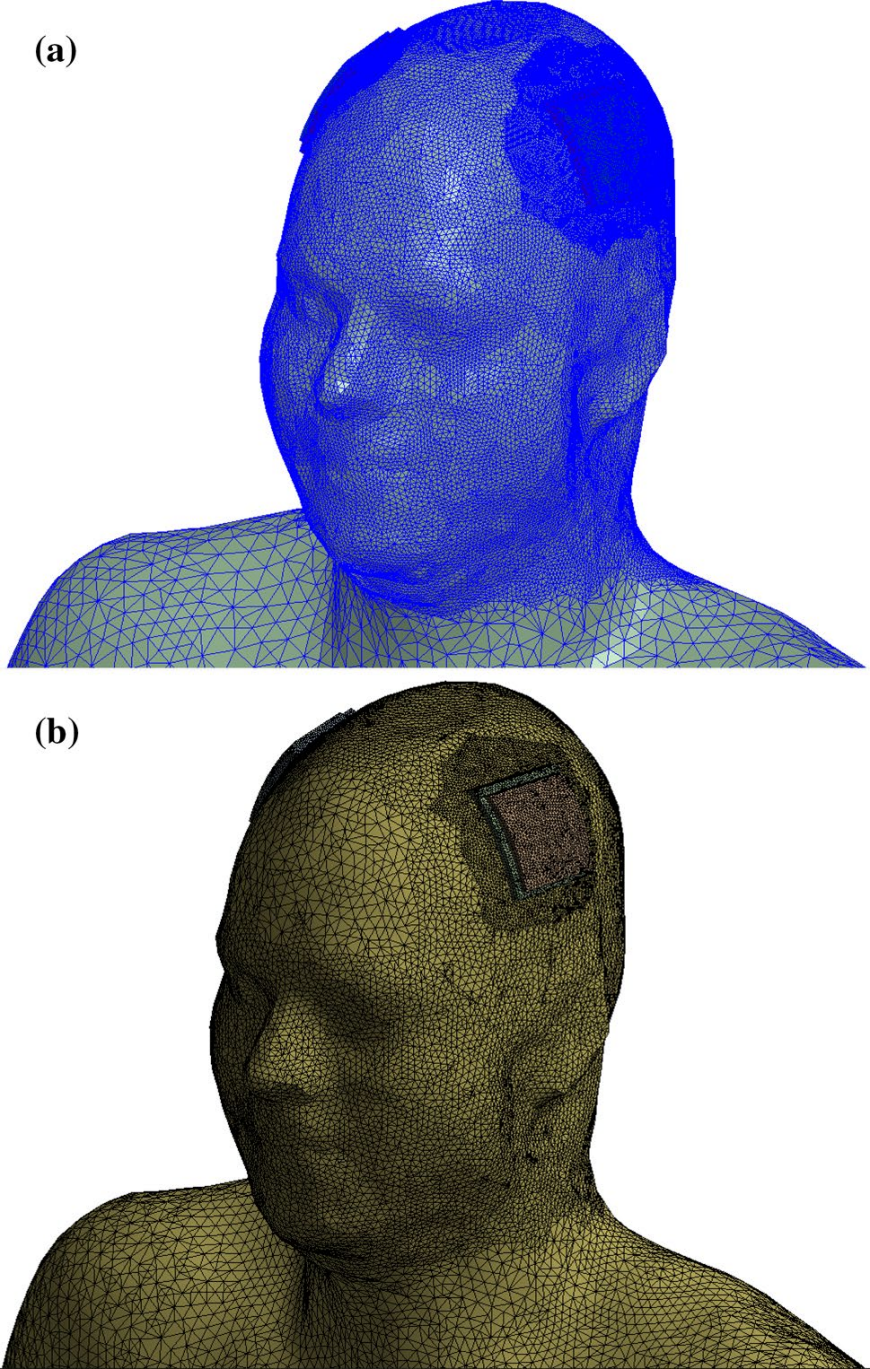
Computation meshes of the human model with tES setup for the **a** 3D EM domain and **b** thermal domain

Tetrahedral elements were used in both the EM and thermal simulations. The computational meshes of the 3D EM and thermal domains were independently generated and adapted in each solver since the EM field and thermal distributions tend to be quite different. This approach ensured the best suitable mesh for each simulation modality (Fig. 4). The power deposited (*PL*_12_) in two L_1_ sub-volumes (*V*_1_*L*_1_ and *V*_2_*L*_1_) centered under both electrodes was estimated during the mesh adaption procedure in ANSYS HFSS using the following equation:

$${{PL}}_{12} = \mathop \int \limits_{ }^{{(V_{1} L_{1} + V_{2} L_{1} )}} \sigma \cdot \left| {E\left( v \right)} \right|^{2} \cdot {\text{d}}v,$$

where *σ* is the electrical conductivity of the L_1_ medium and *E*(*v*) is the electric field distribution. The dimensions of *V*_1_*L*_1_ and *V*_2_*L*_1_ were approximately 70 mm × 70 mm × 4 mm. The volumes of *V*_1_*L*_1_ and *V*_2_*L*_1_ were approximately 20 cm^3^ each. A mesh adaptation procedure in ANSYS HFSS increased the number of mesh elements until the variation of *PL*_12_ between two consecutive meshes was less than 3%. The convergence of the thermal simulations was obtained as in our previous studies [41, 42]. Manual mesh refinement ensured that variations of both the global maximum temperature rise (Δ*T*_max_) and maximum temperature rise in sub-volumes *V*_1_*L*_1_ and *V*_2_*L*_1_ (Δ*T*_VLmax_) were less than 3% for two sequential meshes and that the meshes were refined in the high gradient regions. This procedure resulted in root mean square (RMS) edge lengths in the 3D EM and thermal simulations of: (1) 1.2 mm and 1 mm for the electrodes, (2) 1.1 mm and 0.9 mm for the gel patches, and (3) 1.1 mm and 1 mm for the elements located in layer L_1_ and L_2_ and in close proximity to the electrodes, respectively. The total model, including the whole-body 3T RF coil, tES setup, and human model, consisted of approximately 10 million tetrahedral elements upon completion of the mesh adaption procedure in ANSYS HFSS. The thermal model consisted of approximately 4.5 million tetrahedral elements after refinement in ANSYS NLT. The reduced number of mesh elements in the thermal model is partially due to the reduced number of objects. A comparison of numerical predictions and measurement results that provide a proof of validity of our 3D EM and thermal co-simulation workflow for an MRI coil was recently published [43].

## Results

### Anatomical model results

**Fig. 5:**
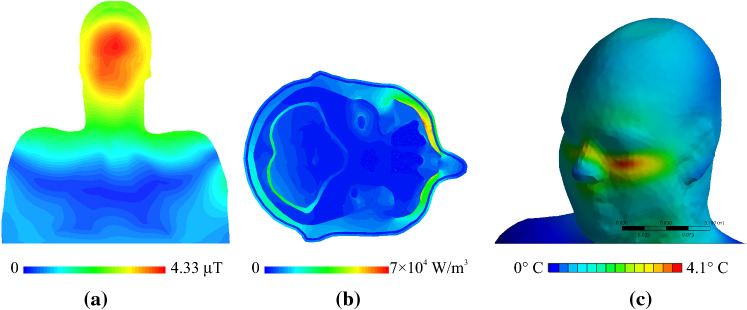
Results for human model only (no tES setup) normalized to 3.2 W/kg headSAR. **a** Coronal profiles of the transverse magnetic field magnetic field component (*B*_1_^+^). **b** Axial profiles of the power deposition at the location of the highest power deposition in the head. **c** Δ*T* distribution on the skin surface

**Fig. 6:**
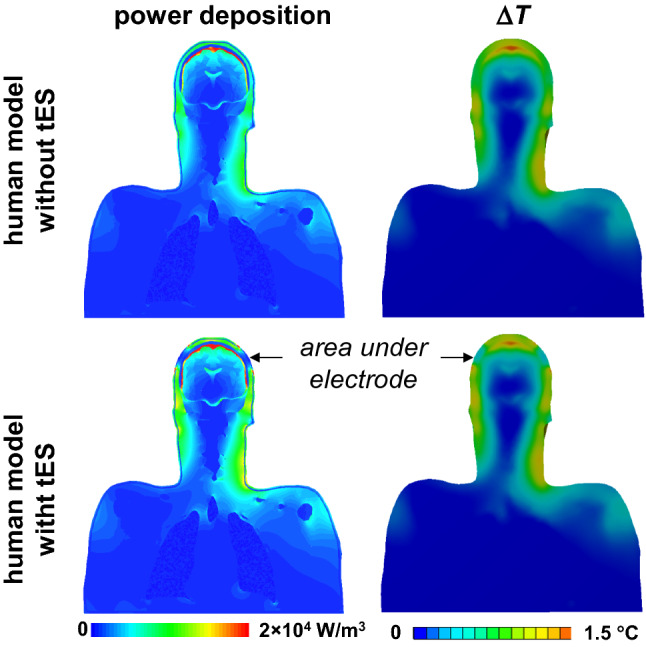
Coronal profiles of power deposition and Δ*T* for the human model with and without tES setup (normalized to 3.2 W/kg headSAR)

As shown in our previous investigation [27], truncation of the human model at the torso resulted in negligible influence on the birdcage coil circuit-level results and field distributions in the head and upper torso. 3D EM results for the model without the tES setup were consistent with common observations in the literature for coronal profiles, see for example [44]. The transverse magnetic field component (*B*_1_^+^) was homogeneously distributed across the head (Fig. 5a) and the maximum deposition of power occurred in the neck region (Fig. 6). The largest power deposition generated by EM exposure from the birdcage coil was located below the left eye (Fig. 5b). This power deposition resulted in Δ*T*_max_ = 4.07 °C after 540 s of continuous exposure (Fig. 5c).

### Results for anatomical models in the presence of the tES setup

**Fig. 7:**
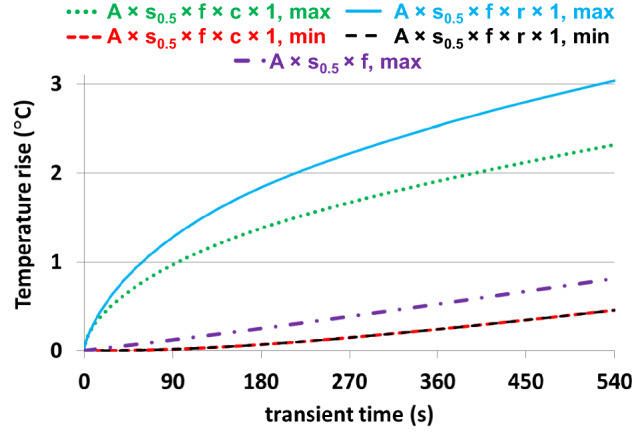
Transient behavior of the minimum and maximum of temperature rises for anatomical models A × s_0.5_ × f × r × 1 and A × s_0.5_ × f × c × 1, i.e., two geometries of gel patches. The temperature rise at the skin location below the electrode in a simulation of the human model without tES, i.e. A × s0.5 × f, is used as a reference

**Fig. 8:**
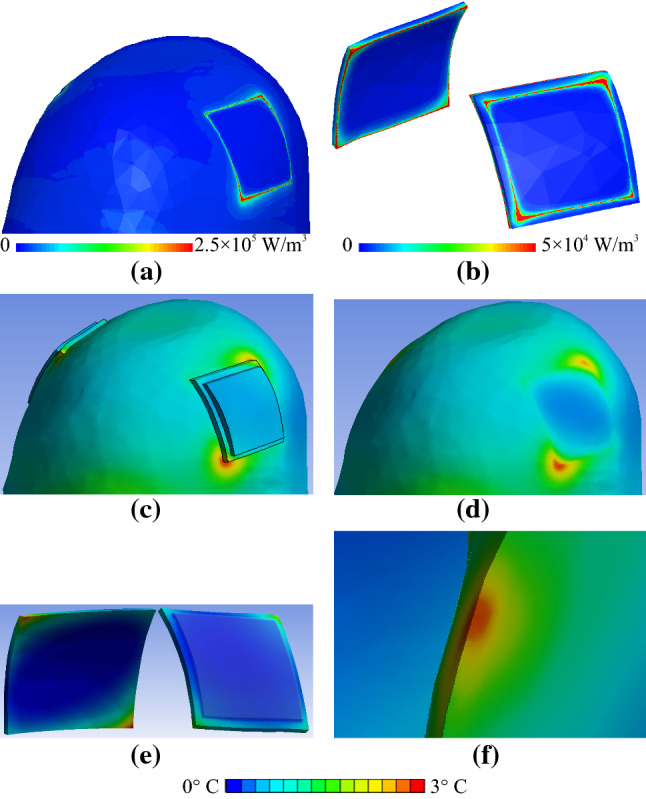
Close-up view of the field distributions in close proximity to the tES electrode after 540 s of RF exposure for anatomical model A × s_0.5_ × f × r × 1. **a** Power deposition in the skin. **b** Power deposition in the gel patches. **c** Δ*T* distribution in the skin, electrodes and gel patches. **d** Δ*T* distribution in the skin (electrodes and gel patches not shown). **e** Δ*T* distribution in the electrodes and gel patches (head not shown). **f** Δ*T* distribution in the head at the location of highest skin Δ*T* and in close proximity to the electrode

**Fig. 9:**
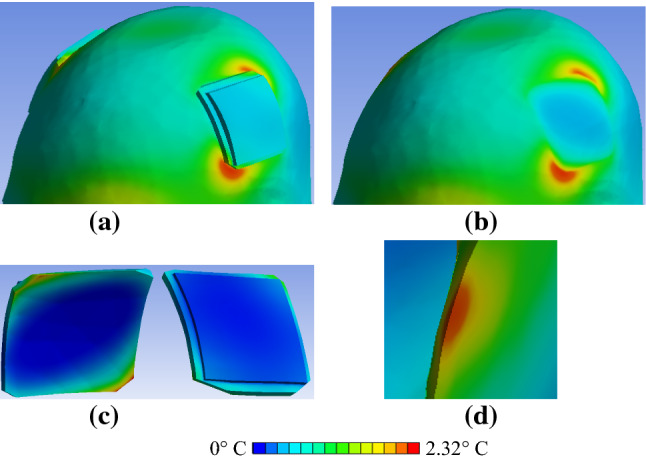
Close-up view of Δ*T* distributions in the human model in close proximity to tES electrode and gel patch with chamfered corners after 540 s of RF exposure for anatomical model A × s_0.5_ × f × c × 1. **a** Δ*T* distribution in the skin, electrodes and gel patches. **b** Δ*T* distribution in the skin (electrodes and gel patches not shown). **c** Δ*T* distribution in the electrodes and gel patches (head not shown). **d** Δ*T* distribution in the head cut at the location of highest skin heating and in close proximity to the electrode

**Fig. 10:**
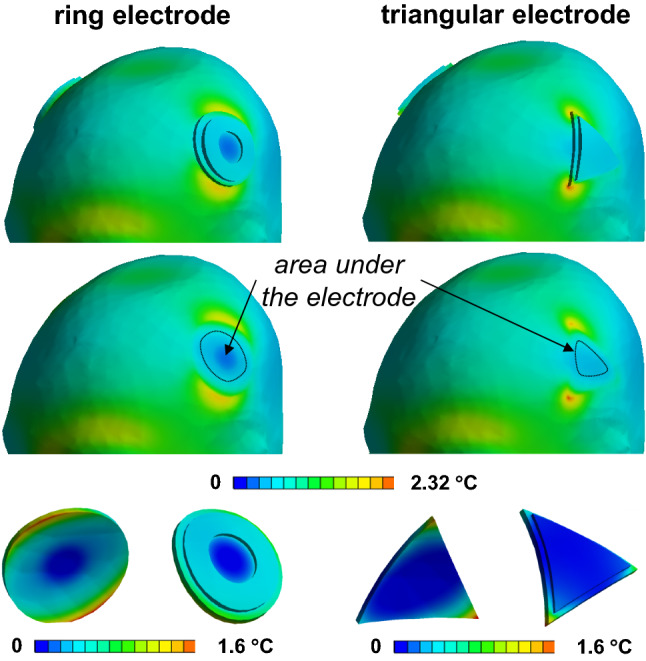
Δ*T* distribution in the skin, electrodes and gel patches for anatomical models A × s_0.5_ × f × i × 1 and A × s_0.5_ × f × t × 1

Including the tES setup in the computational model resulted in small, approximately 3%, decrease of headSAR. Also, the level of power deposition and Δ*T* below the left eye were unaffected. At most locations, Δ*T* of the skin below the electrodes was smaller than Δ*T* of the skin at the corresponding locations in the human model without the tES setup (Figs. 6, 7). This corresponded with a low level of power deposition in the area underneath the electrode. Δ*T* of both the skin and the gel in the area underneath the electrode was up to 10 times smaller than the maximum Δ*T* of the skin and the gel, respectively (Figs. 8, 9, 10).

**Fig. 11:**
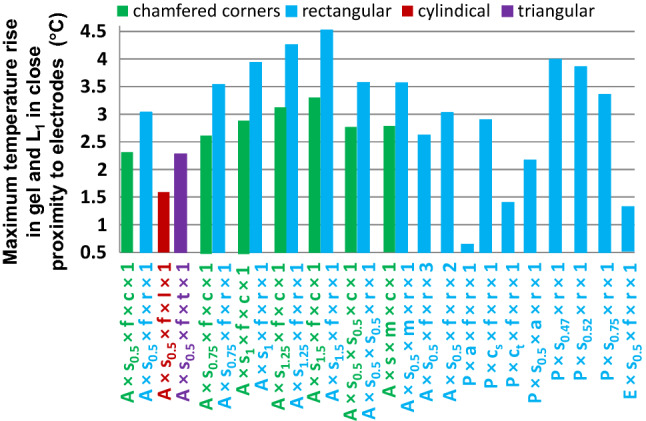
Dependence of maximum temperature rise in the gel and skin, i.e., Δ*T*_VLmax_, in close proximity to electrodes after 540 s of RF exposure

All electrode shapes had significant influence on Δ*T*_VLmax_ (Figs. 10, 11). The hollow cylinder electrode shape had the lowest Δ*T*_VLmax_ (1.6 °C). Varying skin electrical conductivity in the range of 0.5 S/m to 1.5 S/m resulted in increases of ~ 50% in Δ*T*_VLmax_ (Fig. 11) and ~ 22% Δ*T* below the left eye. Changing the material properties of the human model L_1_ and L_2_ resulted in an ~ 20% increase in Δ*T*_VLmax_ (Fig. 11) and less than ~ 3% variation of Δ*T* below the left eye. The relative temperature distribution in the gel patches and electrodes was minimally affected by these changes (~ 5%).

A quantitative comparison of the minimum and maximum transient temperature rise in the gel patches for the rectangular electrodes is presented in Fig. 7. The minimum temperature rise was similar for the two gel patch geometries for square electrodes over the entire exposure time. The high thermal conductivity of the electrodes resulted in very little variation of the maximum electrode temperature after 540 s of RF exposure and the maximum temperature of the electrodes was very close to the minimum temperature observed in the gel patches.

For the rectangular electrodes, substantial variation of power deposition was observed in the skin in close proximity to the tES gel patch edges (Fig. 8a). In areas close to the two opposing patch corners, the power deposition was more than two times greater than the deposition at the other corners (Fig. 8a). The same dependence was observed for power deposition in the gel patches near the electrode corners (Fig. 8b). The highest power deposition in the gel patches was up to fivefold lower than the highest power deposition in the skin. A wide range of Δ*T* (< 1 °C to 3 °C) after 540 s of RF exposure was observed at different locations in the gel and at the skin surface in contact with the gel rectangular patch (Figs. 8c, 9a). The skin area with the highest Δ*T* was not in direct contact with the rectangular gel patch (Figs. 8d, 9b). The maximum Δ*T* was observed in only a small portion of the rectangular gel patch (Figs. 8e, 9c). A substantial influence of the gel patch shape on the temperature rise nearby the electrode and the temperature distribution in skin tissue was also observed (Figs. 8, 9).

The electrical properties of the gel did not have a significant impact on Δ*T*_VLmax_ (variation less than 10%) or the maximum temperature rise in the patches for the rectangular electrodes. In contrast, the sharp thermal gradient in the gel with electrical conductivity of 9.6 S/m was smoother than in the gel with electrical conductivity of 2.2 S/m. For the model without tES wires (i.e., E × s_0.5_ × f × r × 1), Δ*T*_VLmax_ was substantially smaller (i.e., 1.35 °C) than Δ*T*_VLmax_ = 3.05 °C for the model with full tES setup (i.e., A × s_0.5_ × f × r × 1) as shown in Fig. 11.

### Results for experimental phantoms in the presence of the tES setup

Using results for the anatomically accurate phantom as a reference, results for possible experimental phantom setups yielded the following observations (Fig. 11). For P × a × f × r × 1, the maximum Δ*T* in the gel patches decreased by more than 200%, and the acrylic material was heated indirectly from thermal contact with other parts of the phantom. For P × c_s_ × f × r × 1, the temperature rise in the various parts of the phantom was similar to the rise in the human model. For P × c_t_ × f × r × 1, Δ*T*_VLmax_ depended not only on the power deposition in close proximity to the electrodes, but also on the power deposition inside the phantom. As a result, the entire *L*_1_ model was heated to a similar level. For P × s_0.5_ × a × r × 1, Δ*T*_VLmax_ decreased more than 35% relative to A × s_0.5_ × f × r × 1 (Fig. 11).

The following temperature patterns on the gel surface were obtained. The highest Δ*T* was found in areas close to two opposite corners of the gel patch, while a negligible increase of Δ*T* was predicted at the other two opposite corners. The relative temperature distribution in the gel patches showed substantial dependence on the layer material assignment. For example, the already small area with high Δ*T* decreased in P × a × f × r × 1, and the highest Δ*T* was observed at only one corner of the gel patch in P × c_t_ × f × r × 1.

The dependence of Δ*T*_VLmax_ on the electrical conductivity of skin for single-tissue solid gel phantoms was the inverse of that of the anatomical models. Specifically, the increase of skin electrical conductivity resulted in the decrease of Δ*T*_VLmax_ (Fig. 11). Δ*T*_VLmax_ for single-tissue solid gel phantoms was substantially (~ 33%) higher than Δ*T*_VLmax_ for the anatomically accurate phantom.

## Discussion

To the authors’ knowledge, there are no published results that provide thermal results for a similar EM exposure condition, employing a 3T (123.2 MHz) whole-body birdcage RF coil at the head landmark position. In a related study, Wang et al. [45] also observed the largest temperature increase near the eyes, specifically of 1.6 °C. There were several major differences between our models and those of Wang et al. First, Wang et al. used a head-only model that was located in a head coil driven at 64 MHz and 200 MHz. Secondly, his results were only reported for four planes: an axial plane passing through the eyes, an axial plane passing through the center of the coil, and sagittal and coronal planes passing through the center of the coil. Third, Wang et al. used a coarser spatial grid of 3 mm × 3 mm × 3 mm. Finally, blood perfusion was taken into account in his study. Blood perfusion is significantly larger in the region below the eyes than in the scalp, which implies our thermal results include some overestimation of the temperature increase near the eyes. Therefore, temperature increase near the eyes in our human model was used in our sequential analysis only as a relative quantity for assessing the influence of the tES setup.

A major outcome of this study is the significant variation (more than 600%) of Δ*T* in the skin at different locations in close proximity to the electrode. For example, a change of location from one electrode corner to an adjacent corner resulted in more than 400% variation. Thus, significant underestimation of the maximum Δ*T* in the skin can occur if an evaluation of this quantity is restricted to measurements (1) at the surface of the electrodes, (2) underneath the electrodes, or (3) at an arbitrary location inside the gel patches. Previously, temperature measurements for tES applied simultaneously with MRI have only been reported for a setup where “T-type thermocouples were positioned at the skin–electrode interface at both anode and cathode for real-time monitoring of skin temperature” [23]. Δ*T* results presented in that study, for 16 subjects, varied by more than a factor of 10, which serves as indirect confirmation of our modeling observation of large variations of Δ*T* at the skin–electrode interface. It is important to note, however, that the authors did not mention if they tried to position the thermocouples at the same location for all subjects. This is an important consideration since the location of the highest Δ*T* cannot be reliably validated experimentally with a single-probe measurement. Experimental validation of our modeling predictions requires temperature measurements at several locations to reconstruct the spatial temperature distribution (see [46] for an example).

To date, we are not aware of a report of the reliable use of an infrared thermometer or thermal camera for measuring human skin temperature in close proximity to an electrode in an MRI environment. In the presence of a tES setup, accurate measurement of the maximum Δ*T* is challenging even in a phantom study. Δ*T* measurements using luminescence-based sensors includes several uncertainties if a millimetre scale spatial temperature gradient exists. These include the precision of temperature probe locations, the sensitive element sizes of the temperature sensor, and the sensor’s electrical and thermal properties. The smallest tip diameter of an unprotected sensitive element is approximately 0.3 mm. Because such probes must be handled with great care, usually sensors are used that have a sensitive element coated with plastic and an external diameter of more than 0.5 mm. The power deposition is also affected if the dielectric sensor’s tip is located at positions inside the conductive gel. Δ*T* measurements using thermocouples brings additional uncertainties as compared to FOT sensors. For example, the tip diameter of many thermocouples are larger than 1 mm. Thermocouples also include materials that are efficient electrical and thermal conductors, which affects both the power deposition and transient Δ*T* behavior in close proximity to the thermocouple. Therefore, inclusion of the temperature sensor in the numerical domain is required when collecting experimental validation data for Δ*T* modeling.

Our modeling workflow is designed for rapid numerical evaluation of human models located in an MRI RF coil in the presence of a tES setup to estimate the dependence of RF energy-induced heating on different design parameters. One limitation is the neglecting of the influence of blood perfusion, which reduces the local heating of human tissues generated by power deposition. Blood perfusion in the scalp is relatively low compared to the intracranial volume due to its smaller thickness and lower blood-vessel density. Thus, only a moderate skin heating reduction is expected in the vicinity of the tES electrode. An MRI examination can be more than 30 min, which is significantly longer than the 9 min treatment duration modeled in this study. We tried to offset the effect of blood perfusion by applying a relatively short transient heating time.

In a typical implementation of the blood perfusion term in the bioheat equation, *b*(*x*)∙(*T*(*x*) − *T*_b_), where *b*(*x*) is the perfusion coefficient, *T*_b_ is the blood temperature, and *x* is the coordinate of a calculation node. *T*_b_ is often considered to be constant in time and equal to the core body temperature (i.e., 37° C) [37]. Thus, without RF exposure in tissues with temperature equal to or below core body temperature, the perfusion term in the bioheat equation is equal to zero. The thermal balance inside the human model is defined only by metabolic heat and thermal losses from the skin, if the thermal exchange in the lungs is not taken into account. Because metabolic heat was not included in our calculation, we defined the ambient temperature as 37° C to prevent human model cooling due to thermal radiation from the skin in areas with negligible RF exposure levels.

Simonis et al. [46] reported subject-specific RF and thermal simulations and measurements in calf muscle. Using the Pennes’ bioheat equation with blood perfusion, Simonis et al. [46] observed that “the simulations mostly underestimate the temperature increase; the median of the simulations was on average 34% lower than the experimental median” and “the thermal models that were applied in this research were not able to obtain a satisfying match with the experiments, even when subject-specific models were used”. Thus, modeling based on Pennes’ bioheat equation with blood perfusion parameters provided in the literature does not guarantee a satisfactory comparison of measurements and simulations.

It is generally accepted that the skin cannot be segmented from standard 3T MRI in vivo scans. Therefore, the skin and scalp layers were manually added to patient-specific human models from previous studies and the skin thickness varied. Our base value of 2 mm for skin thickness was also used in [46]. The results showed that the skin thickness was an important parameter for Δ*T* modeling in the presence of a tES setup if the material assignment for the second scalp layer corresponded to that of fat. A small influence of skin thickness on Δ*T* was observed if the material assignment for the second scalp layer was not fat. Skin thickness and scalp tissue arrangement can be readily varied in our workflow by defining the required thickness values for L_1_ and L_2_ in ANSYS SpaceClaim and then defining the required material properties for L_1_ and L_2_ in ANSYS HFSS and ANSYS NLT.

The major problems of realistic subject-specific scalp modeling are subject-to-subject variation of scalp layer thickness and material properties, including blood perfusion. Additionally, a lack of tissue material properties for these scalp layers at 123.2 MHz is the reason why modeling a five-layer scalp [47] is not expected to be performed in the near future. Realistic modeling of the human head in the presence of a tES setup is challenging because variation in the skin material properties, in close proximity to the electrodes, interference from hair, etc., can impact the quality of electrical contact with the skin. As one example, the electrical [48] and thermal [49] properties of human tissues are known to be age dependent. However, limited information is available for frequencies around 125 MHz because most previous studies focused on telecommunication frequency bands (500 MHz–2.5 GHz). Indeed, the availability of the required material properties at 123.2 MHz was a general problem in our study. Only the electrical properties of the gel were based on experimentally obtained data, while the material properties of the tES setup were selected from available information in the literature of similar materials.

Since the variation of the gel electrical properties over a wide range of possible values did not result in variation of Δ*T*_VLmax_ larger than 15%, variations of (1) electrode electrical properties and (2) the thickness of the gel patch and electrodes were omitted from this study. However, it cannot generally be concluded that the influence of gel patch thickness and material properties of the gel and electrode on Δ*T*_VLmax_ is always negligible (e.g., for a wide range of electrode designs and dimensions). A more exhaustive statistical study of the dependence of Δ*T*_VLmax_ on scanner type, electrode setup, electrode position, gel thickness, etc. would be required to definitively establish this conclusion.

It is known that tES experiments generate temperature increases in the skin. The level of temperature increase depends on the RMS value of the stimulation current and the electrode dimensions. To cover the above mentioned problems, the safety margin for experiments that include tES simultaneously with MRI is expected to be rather high.

Modeling of various experimental phantoms showed that commonly used phantoms with the enclosure made of a dielectric material (e.g., acrylic) cannot be used for the assessment of Δ*T*_VLmax_ due to the significant material property differences of the enclosure and human tissues. Using an external layer with material properties similar to skin did not solve this problem. 3D printing of a phantom enclosure with material properties similar to skin could also be expensive and time consuming. Therefore, we consider phantoms with dielectric material enclosures good candidates for reverse engineering of MRI coil exposure conditions [50] and validation of the workflow.

Reported results were obtained for a continuous uniform RF pulse that resulted in a headSAR of 3.2 W/kg. In most MRI sequences, RF pulses vary in time and are separated by a period without RF power deposition. The developed workflow can model arbitrary transient MRI pulses or complex groups of MRI sequences. However, modeling an arbitrary transient MRI pulse results in a significant increase of simulation time for transient temperature because the transient time step must be smaller than the time step of the given RF pulse (e.g., order of 10 μs). Using human models [51] and in vivo temperatures measured as a function of time in anesthetized swine [52], it has been shown that the difference between pulse sequences is so small and transient that it should typically be acceptable to consider only the time-averaged SAR in each RF pulse**.** If the thermal time constant of an implant is significantly longer than 10 s (i.e., by more than one order in magnitude), Δ*T* for time-varied RF pulses differs by less than 5% from the Δ*T* for the corresponding continuous uniform RF pulses with the same value of time-averaged SAR [53]**.** For our modeled tES setups, the thermal time constant was more than 150 s (Fig. 10). Thus, a continuous wave represents a good approximation of realistic MRI RF pulses for Δ*T* evaluations. Modeling complex groups of MRI sequences based on time-averaged SAR for each MRI sequence, as was done in [54] for example, does not introduce an increase in simulation time.

This study is only a first step in the complex assessment of RF energy-induced heating. Future research should include, but not be limited to: (1) blood perfusion modeling using the ANSYS parametric design language, (2) temperature probes in the numerical domain for phantom setup modeling, (3) optimization of electrode shape for both tES and RF energy-induced heating, and (4) a coverage of different human subjects, RF coils, and tES setups.

## Conclusions

We presented a computational investigation of RF energy-induced heating, without blood perfusion and metabolic heat, in the presence of a tES setup in an MRI environment using a high-resolution human head and torso model. Simulation results show that the gel geometry, skin electrical conductivity, and position of the temperature sensors have a significant impact (greater than 600%) on the predicted Δ*T*. For measuring the heating of patches in electrical contact with the patient’s skin, the outer layer of a correctly built phantom should be made of a material which closely mimics both the electrical and thermal properties of skin. Because of the difficulty in fabricating such a phantom, future simulation studies will assess the feasibility of a phantom made of acrylic material, with only the area under the patch mimicking the electrical and thermal properties of skin. Furthermore, it is important to know the location of maximum heating (i.e., the hotspot) on the surface of the patch to correctly place the temperature probes. The hotspot can be found using computational modeling or by performing pre-experiments using multiple temperature probes distributed over the device surface. The former is clearly preferred and allows for a more thorough evaluation of the temperature distribution over the subject’s skin surface. Our workflow is targeted at research and development to assess RF energy-induced heating and to understand the nature of the interaction between a tES setup and incident EM fields. The workflow is not expected to be used on its own for device approval. Credible numerical or experimental evaluations, including validation and a thorough uncertainty assessment, should be carried out for each tES setup to assess the RF energy-induced heating.
